# Transcriptional expression of CXCL10 and STAT1 in lupus nephritis and the intervention effect of triptolide

**DOI:** 10.1007/s10067-022-06400-y

**Published:** 2022-11-14

**Authors:** Dongliang Shi, Yan Li, Xiaomei Shi, Meihong Yao, Dan Wu, Yuhui Zheng, Qing Lin, Yinghong Yang

**Affiliations:** 1 Department of Pathology, Fujian Medical University Union Hospital, Fuzhou, China; 2 Department of Breast Surgery, Fujian Medical University Union Hospital, Fuzhou, China; 3 Department of Pain Management, Fujian Provincial Hospital, Shengli Clinical Medical College of Fujian Medical University, Fuzhou, China; 4 Department of Clinical Laboratory, The Affiliated People’s Hospital of Fujian University of Traditional Chinese Medicine, Fuzhou, China

**Keywords:** CXCL10/IP-10, IFN-γ, JAK/STAT1 signaling, Lupus nephritis, Triptolide

## Abstract

**Objective:**

This study screened out the key genes associated with the occurrence and development of lupus nephritis (LN) using bioinformatics methods, and then explored the expression of key genes in LN and the inhibitory effect of triptolide.

**Methods:**

The GEO2R online tool in the GEO database was used to perform differential analysis of gene expression in LN tissues and normal kidney tissues. The GO function and KEGG pathway enrichment analysis of differentially expressed genes (DEGs), STRING, and Cytoscape software were used to build a protein–protein interaction network (PPI) to screen out the Hub gene. Mouse glomerular mesangial cells (MMC) were randomly divided into a control group, an interferon-*γ* (IFN-*γ*) stimulation group, and a triptolide intervention group. The relative expression of CXCL10 mRNA in each group was detected by real-time fluorescent quantitative PCR (RT-PCR). CXCL10 secretion was detected by enzyme-linked immunosorbent assay (ELISA), and Western blot was used to detect the expression of the JAK/STAT1 signaling pathway–related proteins STAT1 and p-STAT1 in each group.

**Results:**

Bioinformatics showed that there were 22 DEGs expression differences in the GEO database. The GO enrichment analysis showed that biological process (BP) such as the type I interferon signaling pathway, innate immune response, IFN-*γ*-mediated signaling pathway, virus defense response, and immune response were significantly regulated by DEGs. Through the combination of String database analysis and cytoscape software, it was found that STAT1 and CXCL10 are closely related to LN. Experimental results showed that IFN-*γ* induces the expression of CXCL10 mRNA and protein by activating the JAK/STAT1 signaling pathway, while triptolide inhibits the expression of CXCL10 mRNA and protein by inhibiting the JAK/STAT1 signaling pathway.

**Conclusion:**

STAT1 and CXCL10 are the key genes in the occurrence and development of LN. IFN-*γ* induces the expression of CXCL10 by activating the JAK/STAT1 signaling pathway, while triptolide inhibits the expression of CXCL10 by blocking the JAK/STAT1 signaling pathway. Inhibition of the JAK/STAT1 signaling pathway and CXCL10 expression is expected to become a potential target for the treatment of LN.

**Table:** 

**Key Points** *• Bioinformatics showed that there were 22 DEGs expression differences in the GEO database.* *• Through the combination of String database analysis and Cytoscape software, it was found that STAT1 and CXCL10 are closely related to LN.* *• Experimental results showed that IFN-γ induces the expression of CXCL10 mRNA and protein by activating the JAK/STAT1 signaling pathway, while triptolide inhibits the expression of CXCL10 mRNA and protein by inhibiting the JAK/STAT1 signaling pathway.*

## Introduction

Lupus nephritis (LN) is an autoimmune disease with renal damage, which is the most common complication of systemic lupus erythematosus (SLE) [1, 2]. LN is pathologically featured by immune complex formation by a large number of autoantibodies, corresponding antigens deposited in microvessels, and complement activation. The basic pathological damage is glomerular cell proliferation, inflammatory cell infiltration, and nuclear fragmentation [3, 4]. In renal biopsies, almost all patients with SLE have varying degrees of renal involvement, and about 5 ~ 20% of patients can develop end-stage renal disease within 10 years, eventually leading to death [5, 6].

Chemokines are small secretory proteins that can mediate the directional migration of immune cells, activate cellular immune activity, and participate in immune regulation [7]. Chemokines and their receptors as a bridge between inflammation and immune response, and they are involved in the occurrence and progression of LN [8, 9]. In recent years, C-X-C chemokine ligand 10 (CXCL10) has been found play an important role in the occurrence and development of LN [10, 11]. CXCL10 is an efficient lymphocyte chemoattractant with biological functions such as activating and chemotaxis of immune cells, mediating inflammation, and inhibiting the formation of new blood vessels [10, 12, 13].

Triptolide (TPL) is an epoxidized diterpene lactone compound extracted from *Tripterygium wilfordii* [14–16]*.* Pharmacological studies and clinical experiments have confirmed that triptolide has immunosuppressive, anti-inflammatory, anti-fertility, and anti-tumor effects [14, 17]. As a monomer drug with great development potential, triptolide has emerged in the treatment of kidney disease [18]. It has been found that triptolide can inhibit the expression of chemokines such as monocyte chemoattractant protein-1 [19]. However, there are no reports linking triptolide with the expression of CXCL10 and STAT1.

In order to explore potential biomarkers and molecular mechanisms in LN, we identified differentially expressed genes (DEGs) between LN specimens and normal kidney specimens. Subsequently, enrichment analysis and PPI networks have found that CXCL10, STAT1, and IFN-*γ*-mediated signaling pathway played important roles in LN. Furthermore, we conducted fundamental experiments to investigate the role of IFN-*γ* and triptolide in LN, which laid a theoretical foundation for triptolide treatment to inhibit LN damage.

## Materials and methods

### Data sets collection

We obtained transcription profile datasets of LN from NCBI GEO databases (http://www.ncbi.nlm.nih.gov/geo/, date last accessed on 6 June 2021). The accession numbers were GSE113342, GSE112943, and GSE32591. The microarray data of GSE113342 based on GPL21847 platforms (nCounter Nanostring Human Immunology v2), GSE112943 was based on GPL10558 platforms (Illumina HumanHT-12 V4.0 expression beadchip), while GSE32591 was based on GPL14663 platforms (Affymetrix GeneChip Human Genome HG-U133 A Custom CDF).

### Identification of differentially expressed genes (DEGs)

The DEGs between LN specimens and normal kidney specimens were identified via GEO2R online tools (https://www.ncbi.nlm.nih.gov/geo/geo2r/, date last accessed on 6 June 2021) with |logFC|≥ 0.585 and an adjust *P* value < 0.05. The intersecting part of the above three datasets was identified using the Venn diagram webtool (bioinformatics.psb.ugent.be/webtools/Venn/, date last accessed on 6 June 2021).

### Enrichment analyses of DEGs

The Database for Annotation, Visualization, and Integrated Discovery (DAVID) v6.8 (https://david.ncifcrf.gov/, date last accessed on 6 June 2021) was used to perform Gene Ontology (GO) annotation and Kyoto Encyclopedia of Genes and Genomes (KEGG) pathway enrichment analysis, which further visualized with R project using a “ggplot2” package [20]. The GO analysis is a common useful method for large-scale functional enrichment research that can be classified into biological process (BP), molecular function (MF), and cellular component (CC) [21]. KEGG is a widely used database which stores a lot of data about genomes, biological pathways, diseases, chemical substances, and drugs [22].

### PPI network construction and hub gene identification

The Search Tool for the Retrieval of Interacting Genes (STRING) [23] (http://string-db.org/, date last accessed on 6 June 2021) database was applied to construct a PPI network for the DEGs and PPI network then visualized by Cytoscape (http://cytoscape.org, date last accessed on 6 June 2021), a general bioinformatics package for visualizing biological network. We further used the MCODE plugin in Cytoscape to analyze the clustering modules and top 10 significant nodes (hub proteins) in the PPI network (ranked by the Degree method).

## Materials

Triptolide was provided by Professor Youwen Lin (Department of Pharmacy, Fujian Medical University); DMEM-F12 medium(#A4192002) and PBS(#10,010,031) were purchased from HyClone Company of the United States, fetal bovine serum (FBS) (#11,011–8611) was purchased from Hangzhou Sijiqing Company. Mouse IFN-γ(#485-MI), AG490(#0414), and CXCL10 ELISA kits(#DY466) were purchased from RD company. CCK-8(#CK04) reagent was purchased from Japan Tongren Institute of Chemistry. The RNA extraction kit(#LS1040), reverse transcription kit(#A3802), and real-time fluorescence quantitative PCR(#A1250) kit were purchased from Promega Company. The upstream and downstream primers of CXCL10 and GAPDH were provided by Shanghai Yingweijieji Company. Rabbit anti-mouse STAT1(#9175S), p-STAT1(#9167S) antibodies were purchased from Cell Signaling Company. Rabbit anti-mouse β-actin(#81,115–1-RR) antibody was purchased from Proteintech Company.

### Cell culture and grouping

Mouse glomerular mesangial cells (MMC)(#GNM21) was purchased from the Shanghai Cell Bank of the Chinese Academy of Sciences. The cells were cultured in DMEM-F12 medium containing 5% FBS at 37 °C in a 5% CO_2_ atmosphere. When the cells grew to 70 ~ 80% confluence DMEM-F12 medium without FBS was substituted for 12 h and used in the following experiments. The MMC in logarithmic growth phase were randomly divided into four groups, a control group, an IFN-*γ* stimulation group, an AG490 intervention group, and a TPL intervention group.

### Cell counting kit-8 (CCK-8) analysis

MMC was planted in a 96-well plate; the number of cells was adjusted to 4.0 × 10^3^/well. When the cells grew to 70 ~ 80% fusion, the DMEM-F12 without FBS was replaced and starved for 12 h. The experimental group was co cultured with cells with different concentrations of TPL (5, 10, 20 ng/ml), and set up blank group and control group. A 10 μl CCK-8 reagent was added after 24 h, and then incubate at 37℃ in 5% CO_2_ incubator for 1 h. The microplate reader reads the OD value while the test wavelength is 450 nm, the reference wavelength is 620 nm. Cell survival rate = (experimental group blank group)/(control group blank group) × 100%。

### RT-PCR analysis

The total RNA was extracted with the RNA extraction kit and then reverse transcribed into cDNA for amplification by real-time fluorescent quantitative PCR (RT-PCR). The primer sequence is shown in Table 1. The total reaction system is 20μL, the reaction conditions are as follows, pre-denaturation at 95℃ for 2 min, 1 cycle used denaturation at 95℃ for 15 s, annealing/extension at 60 °C for 1 min, and 40 cycles were run. GAPDH was used as the internal reference correction and 2^−ΔΔCt^ was used to calculate the relative expression of CXCL10 mRNA. The above experiments were repeated three times.

**Table 1:** Primers of RT-PCR

Name	Forward primer (5′-3′)	Reverse primer (5′-3′)
CXCL10	GCTCAGGCTCGTCAGTTCTAAGT	GGAAGATGGTGGTTAAGTTCGTC
GAPDH	ACGGCAAGTTCAACGGCACAG	GAAGACGCCAGTAGACTCCACGAC

### ELISA analysis

The supernatant from the cell culture in each group was collected and the secretion of CXCL10 protein was detected by enzyme-linked immunosorbent assay (ELISA). The experimental steps were carried out in strict accordance with the instructions of the kit. The above experiments were repeated three times.

### Western blot analysis

The cells in each group were collected, the total protein was extracted by RIPA lysate, and the protein concentration was determined by the BCA method. After protein denaturation proteins were separated by SDS-PAGE gel electrophoresis, transferred to membranes, sealed, and incubated with the first antibody overnight at 4℃. After TBST washing of the membrane they were incubated with the second antibody at room temperature for 2 h then washed with TBST. Membranes were exposed to ECL then developed. The gray value of the band was measured by a gel imaging system. In each experimental group, the formula for calculating the gray value of STAT1 protein was as follows, ratio = (experimental group phosphorylated protein gray value / experimental group total protein gray value) / (blank group phosphorylated protein gray value / blank group total protein gray value).

### Statistical analysis

IBM SPSS version 20.0 (IBM Corp., Armonk, NY, USA) was used for statistical analysis. A Shapiro–Wilk test of normality was performed for all variables. The data of normal distribution were expressed as the mean ± standard error of mean (SEM).The data between two groups were compared by two independent samples T test or two independent samples rank sum test. The data between multiple groups was compared by one-way analysis of variance or multiple independent samples rank sum test, *P* < 0.05 indicated that the difference was statistically significant.

## Results

### Identification of DEGs

**Fig. 1:**
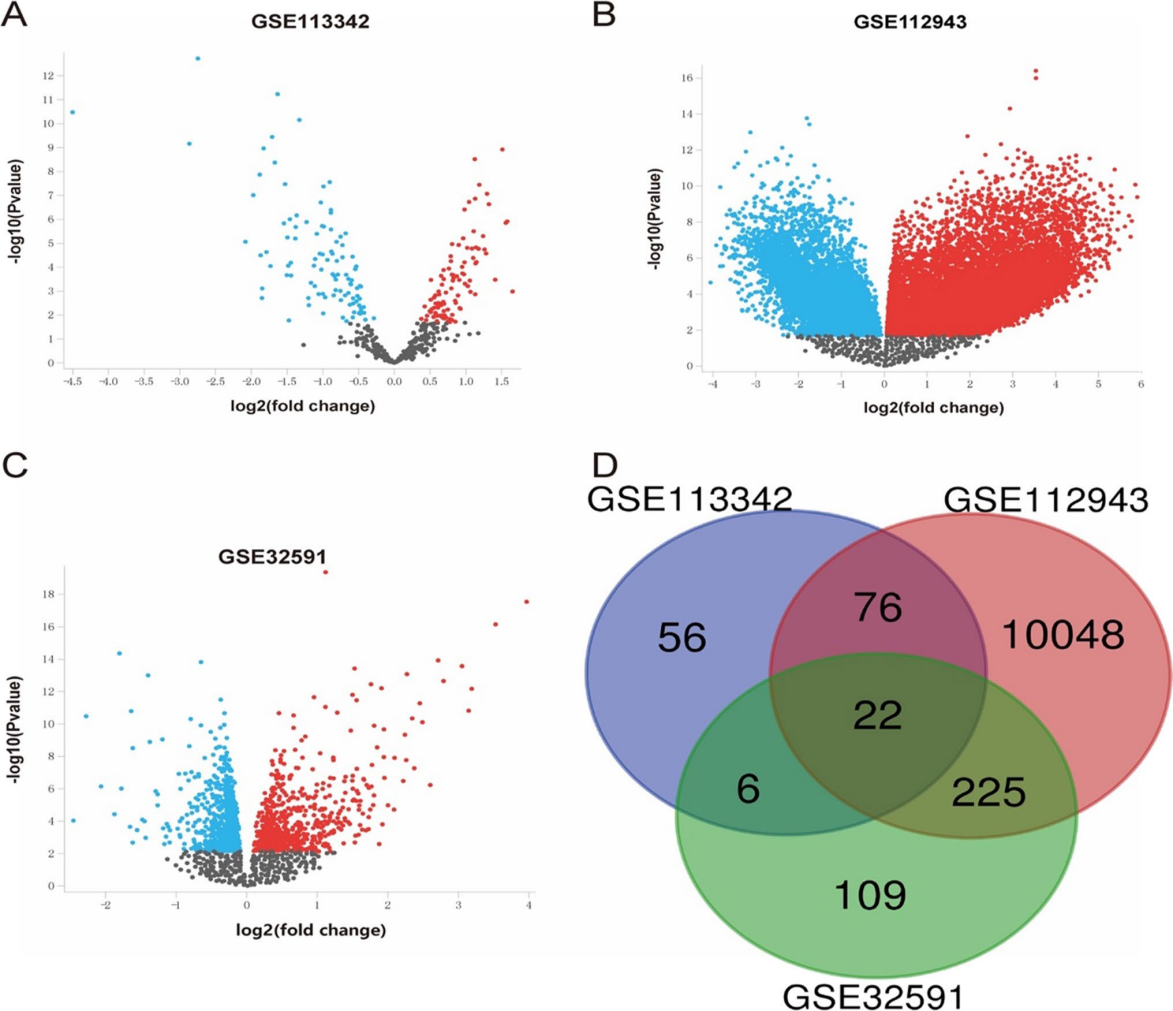
The differentially expressed genes (DEGs) between lupus nephritis (LN) and healthy kidney controls were identified by three transcription profile data (GSE113342,GSE112943, and GSE32591), statistically significant DEGs were defined with |logFC|≥ 0.585 and adjust *P* values < 0.05 as the cut-off criterion

Three gene expression profiles (GSE113342, GSE112943, and GSE32591) were selected in this study. GSE113342 covers 28 LN patients and 16 kidney controls, and GSE112943 contains 14 LN patients and 7 kidney controls, while GSE32591 comprises 64 LN patients and 29 kidney controls, gene expression profiles can be seen in supplementary materials. The DEGs are shown in the volcano plots, subsequently Venn analysis was performed to get the intersection of the DEG profiles (Fig. 1). Finally, only 22 DEGs were significantly differentially expressed among all three groups based on the criteria of |logFC|≥ 0.585 and adjust *P* < 0.05.

### Function enrichment analysis of DEGs

**Fig. 2:**
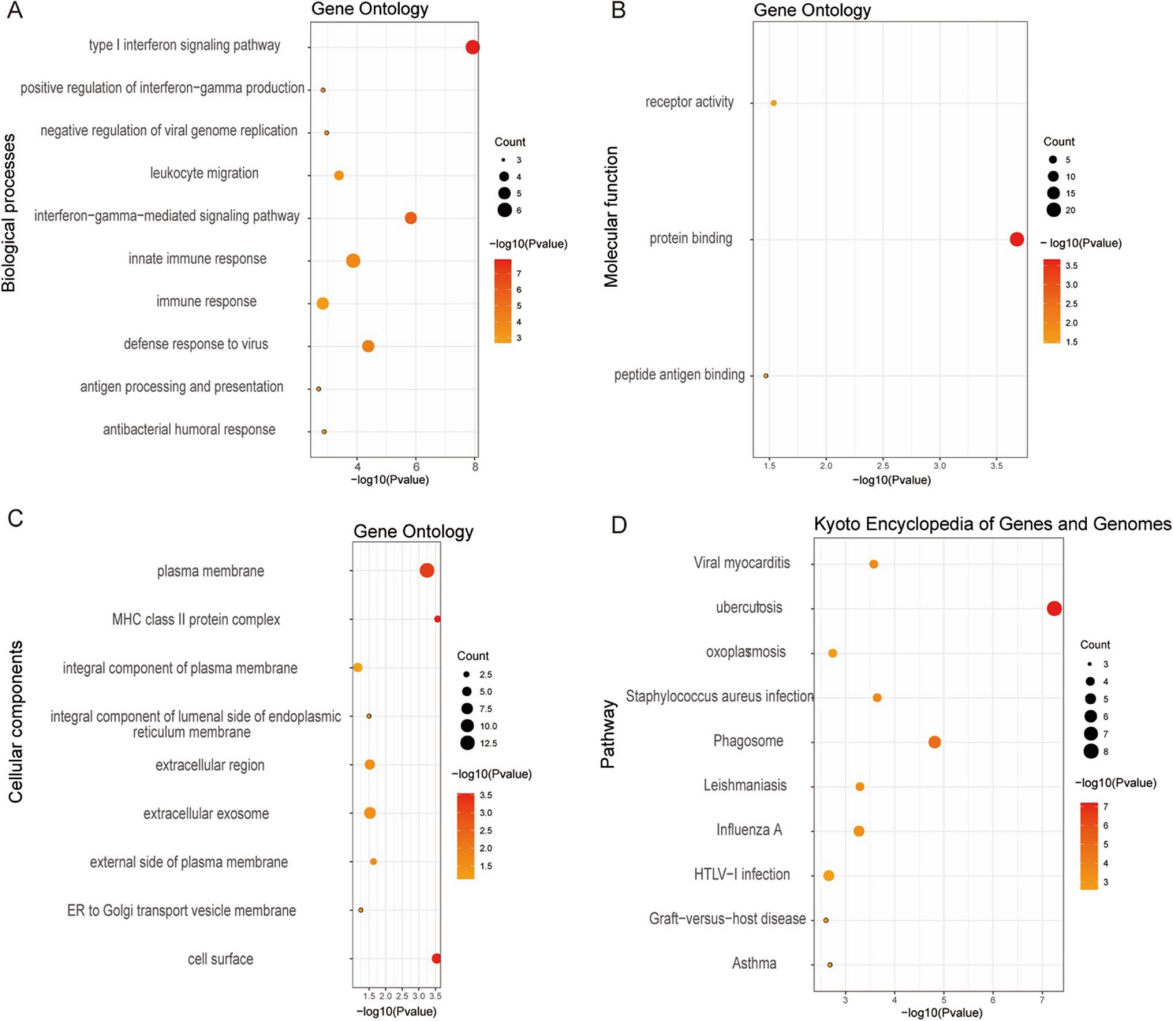
GO functional enrichment analysis on 22 DEGs. **A** Biological process. **B** Cellular components. **C** Molecular functions. **D** KEGG pathway analysis

Presented in Fig. 2 the biological processes such as the type I interferon signaling pathway, innate immune response, IFN-*γ*-mediated signaling pathway, defense response to virus, and the immune response were seen to be regulated by the DEGs. Analyzing molecular functions, we found that the DEGs were significantly enriched in protein binding. For cellular components, the DEGs were mainly focused on plasma membrane, extracellular exosomes, the extracellular region, and cell surface. Among the top 10 KEGG analysis, tuberculosis, phagosome, influenza A, and HTLV-I infection constituted the majority.

### PPI network analysis and hub gene selection

**Fig. 3:**
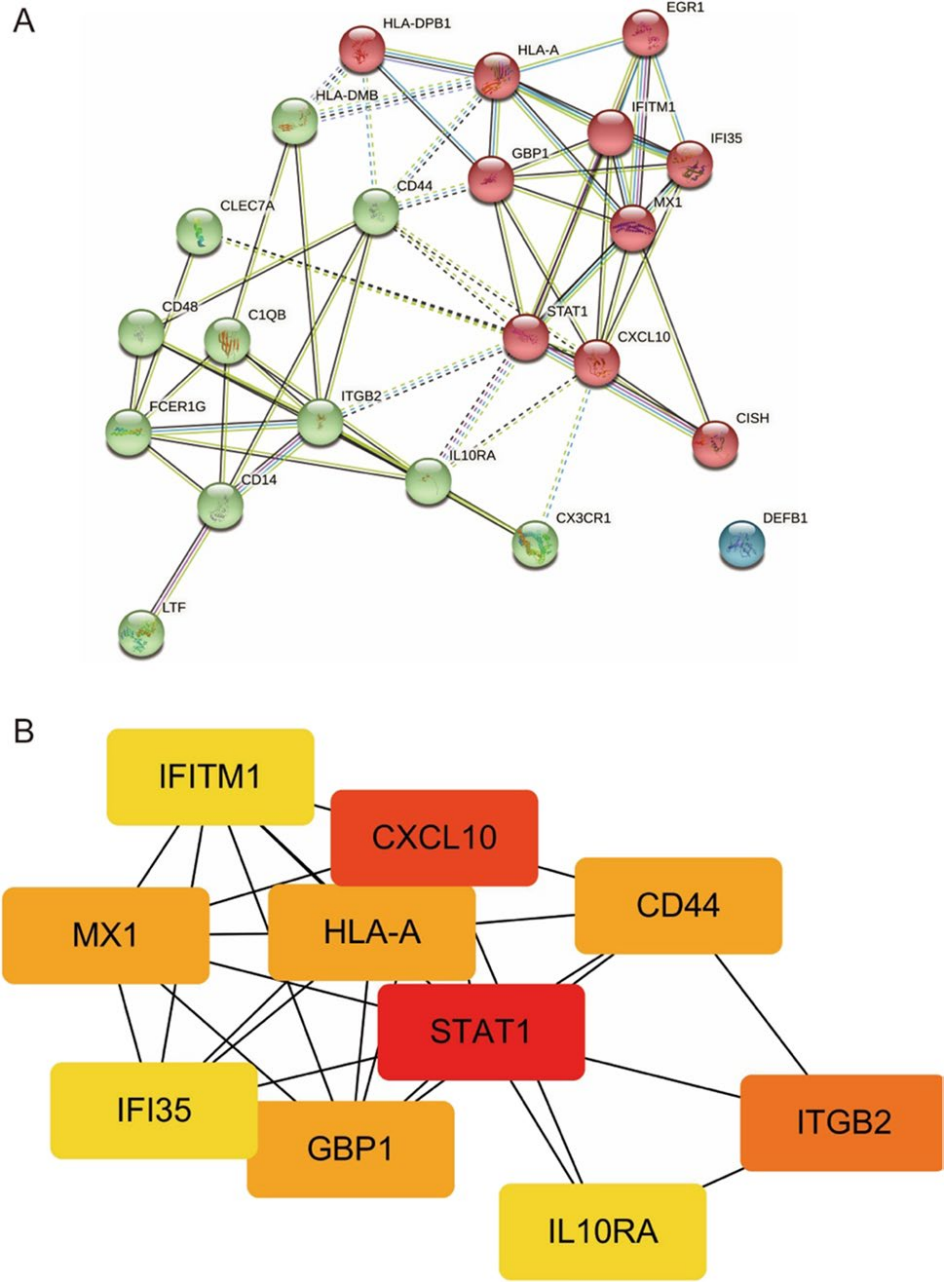
Protein–protein interaction (PPI) network. **A** PPI network of differentially expressed genes (DEGs), **B** subnetwork of top 10 hub genes from the PPI network. Node color reflects the degree of connectivity (red color represents a higher degree, and yellow color represents a lower degree)

As shown in Fig. 3, the PPI network of DEGs, which was based on STRING included 22 DEGs that were gathered as a cluster consisting of 22 nodes and 63 edges. MCODE was applied to identify the most significant module that was comprised of 13 nodes, all downregulated DEGs (MCODE score = 10). The results showed that STAT1 (score = 11) and CXCL10 (score = 10) were the most outstanding genes, followed by ITGB2 (score = 9), MX1 (score = 8), CD44 (score = 8), GBP1 (score = 8), HLA-A (score = 8), IL10RA (score = 7), IFITM1 (score = 7), and IFI35 (score = 7).

### IFN-γ-induced expression of CXCL10 in MMC through the JAK/STAT1 signaling pathway

**Fig. 4:**
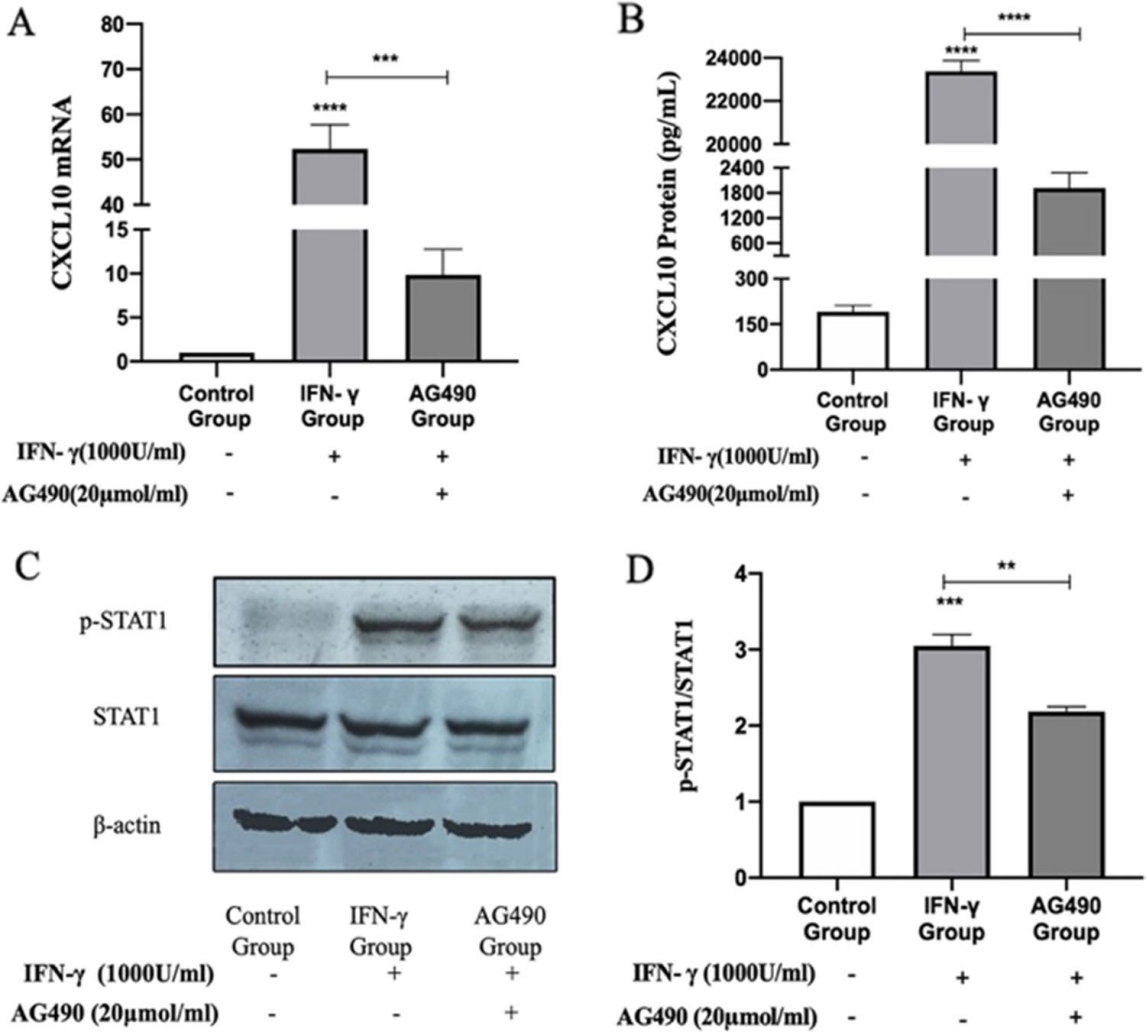
IFN-*γ* induces the expression of CXCL10 by activating the JAK/STAT1 signaling pathway. **A** RT-PCR: the relative expression of CXCL10 mRNA in each group. **B** ELISA: the secretion of CXCL10 protein in each group. **C** Western blot: the expression levels of phosphorylation STAT1 in each group. **D** A summary graph for the densitometry values of the STAT1 protein. (*P < 0.05; ** *P* < 0.01; *** *P* < 0.001)

The IFN-*γ*-mediated signaling pathway, STAT1, and CXCL10 are all related to LN; however, exactly how they work still needs to be understood. To determine the relationship between IFN-*γ*-mediated signaling pathway, STAT1, and CXCL10, we stimulated MMC with IFN-*γ* (1000U/mL) for 24 h then used RT-PCR and ELISA to detect the expression of CXCL10 mRNA and protein. The results showed that there is a low level of basal expression of CXCL10 mRNA and protein in the control group, and after 24 h stimulation by IFN-*γ*, the expression of CXCL10 mRNA and protein was significantly increased (*P* < 0.01). AG490 is a specific blocker of the JAK/STAT1 signaling pathway, and after the treatment of IFN-γ stimulated MMC with AG490, the expression of CXCL10 mRNA and protein decreased significantly (*P* < 0.01), when compared with the IFN-*γ* stimulation group (Fig. 4).

Western blot results showed that after the stimulation with IFN-*γ*, the levels of p-STAT1 in MMC increased significantly when compared with the control group (*P* < 0.01). After treatment with AG490, the level of p-STAT1 in the IFN-*γ*-stimulated cells decreased (*P* < 0.01), as shown in Fig. 4. These results show that, IFN-*γ* induces the expression of CXCL10 by activating the JAK/STAT1 signaling pathway (Fig. 4).

### Triptolide inhibits the expression of CXCL10 through JAK/STAT1 signaling pathway

After intervention with different concentrations of triptolide (5, 10, 20 ng/ml) for 24 h, CCK-8 reagent was added. The results showed that the survival rates of MMC cells were 97.4%, 85.3%, and 67.4% respectively. Triptolide concentration with MMC survival rate > 85% was used in the follow-up experiment.

**Fig. 5:**
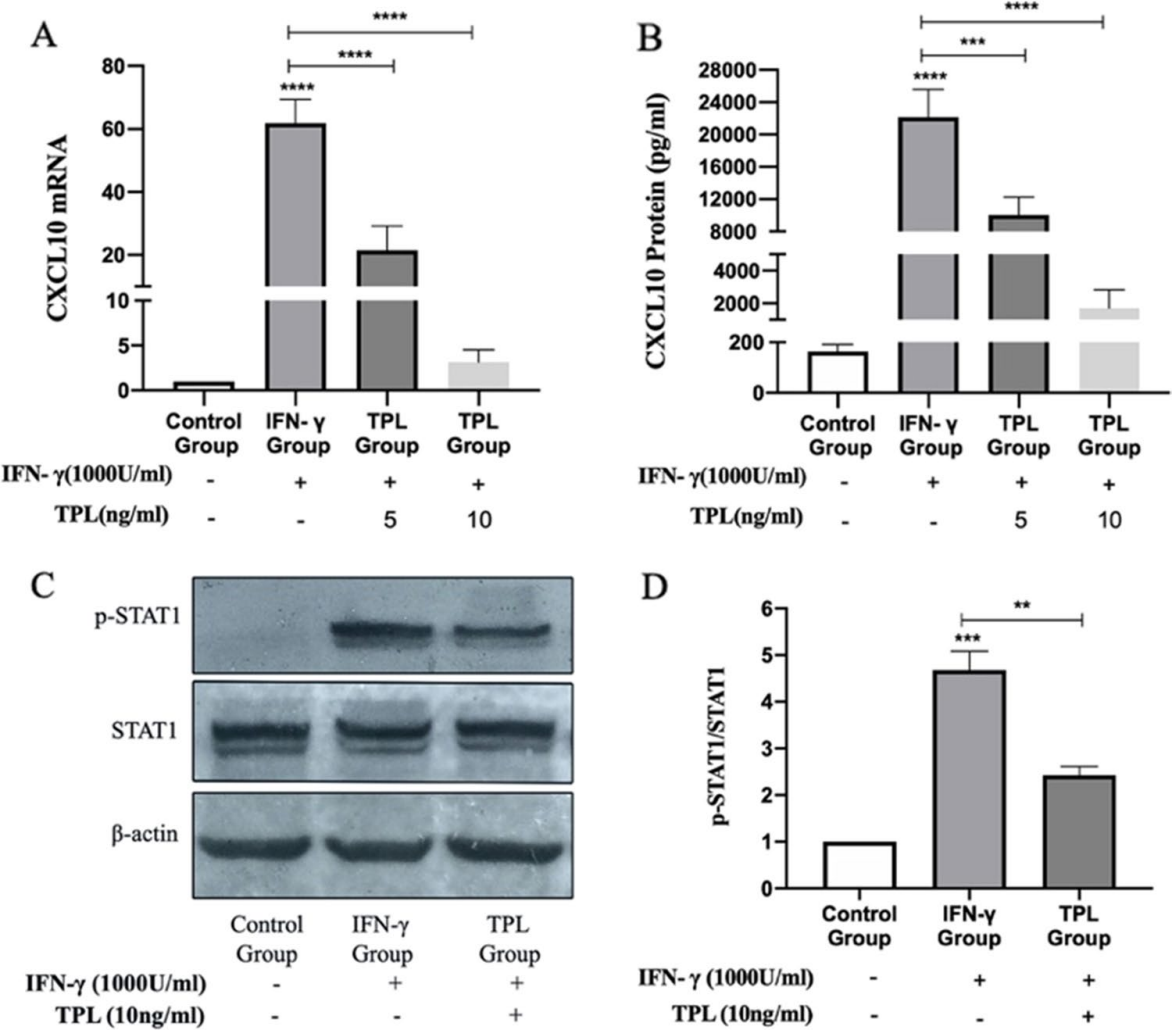
Triptolide reduces the expression of CXCL10 by inhibiting the JAK/STAT1 signaling pathway. **A** RT-PCR: triptolide inhibits CXCL10 mRNA expression. **B** ELISA: triptolide inhibits CXCL10 protein expression. **C** Western blot: The expression levels of phosphorylation STAT1 in each group. **D** A summary graph for the densitometry values of the STAT1 protein. (*P < 0.05; ** *P* < 0.01; *** *P* < 0.001)

To investigate the effect of triptolide on the expression of CXCL10 in MMC, we used two different concentrations of triptolide (5 and 10 ng/ml) to intervene MMC for 2 h, and then IFN-*γ* (1000 U/ml) was added for 24 h. The results showed that after the intervention of triptolide, the expression of CXCL10 mRNA and protein decreased significantly when compared with the IFN-*γ* stimulation group (all *P* < 0.01). These results show that triptolide can inhibit the expression of CXCL10 in MMC that were induced by IFN-*γ* (Fig. 5).

To determine the relationship between triptolide and the JAK/STAT1 signaling pathway, Western blot was used to detect the expression of STAT1 protein and p-STAT1 in each group. After triptolide treatment of MMC for 40 min followed by adding IFN-*γ* (1000U/ml) for 20 min, we found that the levels of p-STAT1 were significantly reduced when compared with the IFN-*γ* stimulation group (*P* < 0.05). These shows that triptolide inhibits the JAK/STAT1 signaling pathway by inhibiting the phosphorylation of STAT1 protein (Fig. 5).

## Discussion

This study analyzed three gene expression profiles in the GEO database (GSE113342, GSE112943, and GSE32591), which included a total of 106 LN patients and 52 control samples. The results showed that there were 22 DEGs expression differences. GO enrichment analysis showed that BP such as type I interferon signaling pathway, innate immune response, IFN-*γ*-mediated signaling pathway, virus defense response, and immune response were significantly regulated by DEG. To further screen out genes that play a key role in the occurrence and development of LN, these 22 differential genes were uploaded to the String database, and a protein interaction network consisting of 22 points and 63 edges was constructed. Through visualization in MCODE, it was found that STAT1 and CXCL10 are most closely related to LN.

Glomerular mesangial cells are the main pathological site of LN [24–26]. There are different degrees of proliferation of glomerular mesangial cells in kidney biopsies of patients with LN, and a large amount of immune complexes are deposited in mesangial cells [3, 5, 27]. IFN-*γ* plays an important role in the occurrence and development of LN. Yazici et al. [28] found that the level of IFN-*γ* in LN patients was higher than that in healthy people; patients with tumors or liver diseases could induce SLE after receiving IFN therapy [29]. In animal experiments, it was found that IFN-γ can promote the production of autoantibodies [30, 31]. Hayakawa et al. [32] pointed out that IFN-*γ* enhances mesangial cell activation through inflammatory stimulation, increased deposition of adhesion molecules and immune complexes, and cell migration. Especially in early LN, IFN-*γ* may contribute to the exacerbation of IMQ-induced LN by promoting early leukocyte infiltration into the kidney. These results suggest that IFN-*γ* is involved in the occurrence and development of LN. In view of the above results, we took MMC as the research object to clarify the relationship between IFN-*γ*, STAT1, and CXCL10. We used RT-PCR, ELISA, and Western blot to clarify the expression of CXCL10 mRNA and protein, and the expression of STAT1 and p-STAT1 related to the JAK/STAT1 signaling pathway.

Our results show that IFN-*γ* induces the expression of CXCL10 by activating the JAK/STAT1 signaling pathway. This result has also been reported in other studies. Aota et al. [33] found that IFN-*γ*-induced CXCL10 expression in human salivary gland ductal cells through the JAK/STAT1 signaling pathway, Han et al. [34] conformed RAW264.7 cells could express CXCL10 when stimulated by IFN-*γ*. Similarly, cepharanthine suppresses IFN-*γ*-induced CXCL10 production via the inhibition of the JAK2/STAT1 signaling pathway in human salivary gland ductal cells [35]. It can be seen that IFN-*γ* activates the JAK/STAT1 signaling pathway and then induces an increase in the expression of CXCL10. CXCL10 is a highly potent lymphocyte chemotactic agent, it may be involved in the occurrence and development of LN by activating and chemotactic immune cells to renal tissue, IFN-*γ*, tumor necrosis factor-*α*, etc. to participate in inflammatory responses, inhibiting the formation of new blood vessels, and reducing the activity of antibiotics [10, 11, –13]. In animal experiments, it was found that inhibiting the JAK/STAT signaling pathway can reduce the expression of anti-dsDNA, reduce the production of urinary protein, improve splenomegaly and reduce the level of kidney inflammation in NZB/NZW F1 mice [36]. In a phase I double-blind randomized safety clinical study, short-term use of the JAK/STAT signaling pathway inhibitor tofacitinib in subjects with mild-to-moderate SLE was found to be generally safe and well tolerated with no surprises adverse events, thromboembolic events, or opportunistic infections [37]. In conclusion, by inhibiting the JAK/STAT1 signaling pathway to reduce the expression of CXCL10, it can reduce inflammatory cell infiltration, reduce or block the inflammatory response, and is expected to become a potential treatment for LN.

With the use of hormones combined with immunosuppressive agents in the treatment of LN, the survival rate of LN patients has been significantly improved, but still has adverse reactions such as serious side effects and secondary infections [38]. Therefore, it is important to find new therapeutic drugs. Both basic experiments and clinical trials have confirmed that triptolide have good curative effects on SLE and LN. Triptolide is one of the effective components of *Tripterygium wilfordii* polyglycosides [14, 39, 40]*.* It has a variety of pharmacological effects such as immunosuppression, anti-inflammatory, anti-fertility, and anti-tumor effects [14, 41]. In this study, we found that triptolide inhibits CXCL10 expression by inhibiting the JAK/STAT1 signaling pathway. In animal experiments, Zhao et al. [42] found that triptolide can improve serum anti-dsDNA, proteinuria, and renal histopathological evaluation in MRL/lpr mice. Tao et al. [43] found that triptolide can improve the survival rate of mice with LN, reduce the severity of the disease, and reduce the production of cytokines. In our experiment, triptolide inhibited the expression of CXCL10 in MMC and the expression of JAK/STAT1 signal pathway–related proteins was also inhibited. Therefore, we believe that triptolide inhibits the expression of CXCL10 by inhibiting the JAK/STAT1 signal pathway. The ability to inhibit the expression of CXCL10 in glomerular mesangial cells may make triptolide a potential agent for the treatment of LN.

This topic not only provides new ideas for the mechanism of LN, but also lays a theoretical foundation and provides experimental data for the application of triptolide in the clinical treatment of LN. However, this study has some limitations, such as insufficient mechanistic studies, lack of animal experiments, and the fact that cellular models do not represent the true condition of the disease. The next step we are going to do animal experiments and use gene editing to verify key targets, lay the foundation for the application of triptolide in the treatment of LN.

## Conclusion

In this study, we obtained information about the important role of STAT1 and CXCL10 in LN through bioinformation analysis. Basic experiments confirmed that IFN-*γ* induces the expression of CXCL10 through the JAK/STAT1 signaling pathway, and triptolide inhibits the expression of CXCL10 through the inhibition of the JAK/STAT1 signaling pathway.
